# Weakness and the Inability to Ambulate in a 14-Month-Old Female: A Case Report and Concise Review of Guillain-Barre Syndrome

**DOI:** 10.1155/2013/953612

**Published:** 2013-01-30

**Authors:** Scott A. Bloch, Mahsa Akhavan, Jahn Avarello

**Affiliations:** ^1^Department of Emergency Medicine, SUNY Upstate Medical University, Syracuse, NY 13210, USA; ^2^Department of Emergency Medicine, Medical College of Georgia, Augusta, GA 30912, USA; ^3^Department of Pediatrics, Division of Pediatric Emergency Medicine, Cohen Children's Medical Center, New Hyde Park, NY 11040, USA

## Abstract

Guillain-Barre syndrome (GBS) is an acquired disease of the peripheral nervous system which causes demyelination and leads to weakness, ataxia, and areflexia. There are a variety of forms of the syndrome, and although it is found in all age groups, it is rare in children less than two years of age. The present complaint of weakness, ataxia, or lower extremity pain in the pediatric population should cause the practitioner to consider GBS in the differential. We describe a case of a 14-month-old girl presenting with weakness and the inability to ambulate who was diagnosed with GBS. The purpose of this paper is to review the emergency medicine diagnosis and management of Guillain-Barre syndrome in children.

## 1. Case

 A 14-month-old female was brought to our pediatric emergency department by her father who stated that the child would not walk. This refusal to walk was noticed by the parents twenty-four hours prior to presentation in the ED. Before that time, the child was walking normally. She had no history of developmental delay and ambulated easily and commonly ran without difficulty. In the ED, the parents reported that the weakness was progressing and the child was now even having difficulty crawling. They thought that the left leg was worse than the right and that she was also having difficulty grasping objects, again more so with her left hand. 

 The child was diagnosed with pneumonia three weeks prior to presentation to our ED and was treated with Cefdinir. Otherwise, her past medical history was unremarkable. She received varicella, hepatitis A, and influenza vaccinations four days prior to her ED presentation. On review of systems, the patient had not had a bowel movement for 36 hours, when normally she had a daily bowel movement. The family also denied any fevers. Immediately prior to the observed weakness, the child had been in the care of a babysitter; however, no history or signs of trauma were present. The child had no allergies, was not on any medications, was born at term with no complications, and did not have any past surgeries. The child lives with her mother, father, and four siblings. The only pertinent family history was a maternal grandmother with multiple sclerosis.

 In the emergency department the child was smiling and was interacting appropriately. On examination, her temperature was 37.7, blood pressure 88/palpable, heart rate 150, respirations 20, oximetry 100% on room air, and weight 10.3 kg. Her head was atraumatic and normocephalic, and extraocular movements were intact, pupils were equal, round, and reactive to light and accommodation. Neck was supple, no lymphadenopathy was palpated, face was symmetrical, and tympanic membranes were normal bilaterally. There was no erythema, exudates, or other abnormalities observed in the oral cavity. Lung, heart, and abdominal exams were normal and unremarkable. Extremities were warm, pulses were normal, and capillary refill was less than two seconds. No bruises or rashes were observed on the skin. Genitourinary exam was normal.

 Although, on neurological examination, the child was moving all four extremities spontaneously, she would not bear weight in order to stand or to walk. There was no bony tenderness or deformities on palpation of the long bones. When positioned on her stomach to crawl, the left lower extremity was moving noticeably less than the right lower extremity and was much weaker than the right when her strength was tested. Both lower extremities, however, were weak on exam. While on her back, we pushed her thighs against the table and tickled the child to see if she would move her legs. This resulted in lifting of her right leg slightly but not the left leg. The right grip was slightly stronger than the left; however, when given a toy, the child's grips were so weak that the toy fell to the exam table from both hands. Both upper and lower extremity reflexes were absent. The child was able to hold her head up without difficulty.

 A BMP (basic metabolic panel), CRP (C reactive protein), and LFTs (liver function tests), which were obtained during her emergency department stay, were all normal. A head CT performed after sedation with 1 mg versed and 2 mg morphine was normal with no fluid collections and no midline shift. A lumbar puncture performed after consent was obtained from the parents resulted in clear CSF (cerebrospinal fluid) with no red blood cells and 2 white blood cells (100% monocytes). CSF glucose was 64 mg/dL, and protein was 25 mg/dL. No organisms were seen on the gram stain, and CSF cultures had no growth after 3 days. 

 The patient was admitted to the pediatric floor after a neurology consult. While in the hospital, an EMG study showed no evidence of myopathy or longstanding axonal loss. It also revealed slow proximal conduction velocities and absent F waves. These EMG results were consistent with an acute demyelinating neuropathy such as GBS. Antinuclear antibody testing and complements C3 and C4 were all normal. The patient was diagnosed with Guillain-Barre syndrome and given a full five-day course of IVIG. 

 During her week-long stay in the hospital, the patient's upper and lower extremity strength significantly improved. She had no respiratory problems during her hospital course. The patient was discharged seven days after her ED presentation with near complete resolution of the weakness. She continued to have difficulty in making a fist. 

 Followup in the neurology clinic one month after her initial presentation to the ED showed little evidence of GBS. The mother stated that about one week after discharge the child was crawling. This quickly progressed to pulling up, cruising, and then the child was walking, though initially very gingerly. The only residual symptom that the mother still notices is that, with prolonged walking, the child's right foot will turn in after a time. Occupational therapy did not feel that she required any ongoing therapy. She has not been sick, is doing well at home, and the family has no other complaints. Exam was grossly normal with normal upper and lower extremity strength, gait, and pincer grasp. Deep tendon reflexes in her biceps and brachioradialis were barely elicited and were questionable in her knees. However, the clinic note stated that her cooperation was suboptimal and the results of the deep tendon reflexes were questionable. The impression was that the Guillain-Barre syndrome was to a large degree clinically resolved and the child could followup with her pediatrician.

## 2. Discussion

### 2.1. Introduction

 Guillain-Barre syndrome (GBS) is a clinical syndrome that is most commonly recognized by progressive weakness of the extremities and loss of deep tendon reflexes. The syndrome occurs in men more than women and in both the adult and pediatric population. The progression of the weakness can occur within days to a month, and usually spontaneous remission of the weakness and paralysis occurs. The mechanism involving the pathophysiology varies according to the form of the syndrome, but is autoimmune in nature. The purpose of this paper is to review the emergency medicine diagnosis and management of Guillain-Barre syndrome in children.

### 2.2. Epidemiology

 It is difficult to ascertain the actual incidence of GBS because there is no available test for diagnosis. GBS occurs worldwide and in all age groups, but peaks during early adulthood and in the elderly. The incidence is 0.5–1.5 cases per 100,000 population in individuals younger than 18 years [1]. However, the syndrome is rarely observed in infancy and is uncommon in children less than two years of age. One pediatric study of 61 GBS cases from Argentina between 1994 and 1996 showed a range of 14 months to 14 years [2].

### 2.3. Etiology and Pathology

 GBS is preceded in two out of three cases by an illness, most commonly gastroenteritis or a respiratory tract infection [3]. This illness resolves prior to the onset of the neurological symptoms. It is believed that the syndrome is due to an immune response, which includes inflammation and antibody production, to a preceding infection. 

*Campylobacter jejuni* and *Mycoplasma pneumoniae* are the most common bacterial infections, while cytomegalovirus, varicella zoster, and Epstein-Barr are the most common viruses associated with the disease [4]. 

 Features of the clinical course of GBS can differ based on the infectious process which preceded the development of the neurological symptoms [5, 6]. Specific antibodies formed to the preceding infection cross-react to the patient's own nervous tissue, resulting in a GBS type specific to that disease. This is known as cross-reactivity or molecular mimicry. One form of *C. jejuni*, for example, has an epitope which causes an antibody response in the patient. These specific antibodies recognize and bind to areas on the oculomotor, trochlear, and abducens nerves leading to the symptoms found in Fisher syndrome [7]. 

 Many studies have attempted to show an association between vaccinations, particularly the influenza vaccine, and GBS; however, much debate remains regarding this topic [8–12]. Though it is possible that there is a link between GBS and vaccinations, more studies are needed.

### 2.4. Clinical

 GBS is the most common acquired polyneuropathy observed in the acute setting in the pediatric population. The average age is 6.3 years old, but ranges from 11 months to 17.7 years [13]. Very young children who cannot verbalize as clearly as older children or adults can be more difficult to diagnose. Presentations in the young pediatric population include weakness, refusal to walk, and limping [14]. About nine out of ten cases have asymmetrical weakness with the remaining cases having symmetrical weakness. Pain can also be a main complaint [15]. Therefore, lower extremity pain combined with the loss of deep tendon reflexes should raise concern for GBS in the differential diagnosis. Although GBS is more benign in the pediatric population, there is the potential for a poor outcome if not diagnosed. 

### 2.5. Variants

 There are a variety of types of GBS that have been described. These include acute inflammatory demyelinating polyneuropathy (AIDP), acute motor axonal neuropathy (AMAN), acute motor and sensory axonal neuropathy (AMSAN), and the Miller Fisher syndrome (MFS). In North America, Europe, and Australia, about 85% of GBS is of the AIDP type [4], while AMAN is the most common in China, Japan, and South America [2]. A study of Mexican children showed that both AMAN and AIDP forms of GBS were common [16].

 AIDP typically causes an ascending paralysis, involving the sensory nerves with loss of deep tendon reflexes. There are continual fluctuations in pulse and blood pressure as well. Resolution of symptoms occurs with repair of the demyelinated sections and can take longer than six months. If symptoms continue for over two years, full recovery is not expected. The amount and severity of the axonal loss correlate to the disease severity and the final long-term disease outcome [17]. 

 AMAN only affects the motor aspect of the nervous system and most of the cases do not involve the autonomic nervous system. Recovery time in patients with AIDP and AMAN is similar. The prognosis is usually good.

 AMSAN involves sensory nerves but is otherwise similar to AMAN. This form is usually more severe, has a longer period of recovery, and is an uncommon form of the syndrome [18].

 The Miller Fisher syndrome (MFS) is another form of GBS. The clinical features of this type, including ataxia, ophthalmoplegia, and areflexia, are related to the specific epitopes recognized by antibodies on cells in the dorsal root ganglion, oculomotor nerves, and neurons in the cerebellum, respectively. The entire disease process is thought to be started by specific strains of *C. jejuni* [6].

### 2.6. Differential

 The differential diagnosis of GBS is broad. Botulism, transverse myelitis, compressive myelopathy, poliomyelitis, toxins, tick paralysis, diphtheria, porphyria, myasthenia gravis, bilateral cerebral strokes, and polymyositis are some of the main considerations [19, 20].

 Botulism is a food-borne disease. There is often a history of eating canned foods or honey in infants less than one year of age. Common findings include ptosis, dilated pupils, pupils that respond poorly to light, and blurred vision. Other distinguishing features from GBS include normal reflexes, a descending paralysis, and constipation. Also, if there is no cranial involvement, this virtually rules out the diagnosis of botulism. 

 Transverse myelitis due to inflammation of the spinal cord bilaterally at the same level is thought to have an autoimmune component. The result is destruction of the myelin. It can occur at all levels of the spinal cord and affects all levels below the affected area. The common presentation is leg weakness, paralysis, back pain, urinary retention, and loss of bowel control. The most common area affected is the thoracic area. MRI of the spinal cord at the level indicated by exam is required for diagnosis. 

 Compressive myelopathy causes destruction of spinal cord tissue due to a mass. This can be a tumor, hematoma, abscess, or degenerative spine disease. The symptoms are similar to those of transverse myelitis and can include pain, numbness, paresthesia, and motor disturbances. These symptoms will again be related to the level and degree of compression on the spinal cord.

 Polio myelitis is rare due to the polioimmunization, but it is still seen in immunocompromised individuals. It causes a weakness or paralysis that is usually asymmetric with loss of deep tendon reflexes. Bowel and bladder function usually remain normal. The symptoms are preceded by a febrile illness.

 Toxins included in the differential of GBS include heavy metal intoxication in pediatrics, glue sniffing of hexacarbons, and organophosphate poisoning. All of these can cause signs of an acute peripheral neuropathy. In the pediatric population encephalopathy is almost universally present with heavy metal poisoning. Symptoms vary greatly and can include neurological and GI symptoms. In the adolescent population glue sniffing should be considered in the differential. The hexacarbons in glues and lacquers are neurotoxic. Organophosphate poisoning can cause a toxic neuropathy. The effects can be remembered by the common mnemonic SLUDGE, which stands for salivation, lacrimation, urination, diaphoresis, gastrointestinal motility increased, and emesis.

 Tick paralysis often has a history of a tick bite, or a tick is found in the hair on exam. Removal of the tick leads to resolution of symptoms. Diphtheria can lead to a severe polyneuropathy secondary to demyelination but immunization status typically rules out this cause. 

 Porphyria is a metabolic disorder affecting heme biosynthesis that mainly affects the peripheral nervous system and the skin. It does occur in children but is very rare. 

 Myasthenia gravis is usually a gradual disease process and does not present as an acute weakness. Reflexes are preserved, and ptosis and ophthalmoplegia are almost always present. 

 A bilateral cerebral stroke is extremely rare but could cause bilateral paralysis and/or weakness. Polymyositis causes muscle inflammation and leads to weakness of the proximal limb muscles occurring over weeks to months. It is more common in women and mainly affects adults in their fifth or sixth decade of life.

### 2.7. Diagnosis

 There is no diagnostic test to confirm the presence or absence of GBS at this time, and the variants of the syndrome most likely have different pathophysiologies. Diagnosis, however, requires areflexia and progressive weakness in the upper and lower extremities. Other indicators of the disease include electrodiagnostic abnormalities, dysfunction of the autonomic nervous system, and elevated CSF protein [21]. The elevated CSF protein levels are typically not present until two weeks after the initial symptoms present. 

### 2.8. Treatment

 Treatment includes supportive care and treating the immune response associated with the syndrome. Appropriate nursing staff in an intensive care setting is essential. A monitored floor bed in a facility with an ICU/PICU present is an option for mild cases. These include patients that lack respiratory involvement, autonomic instability, or weakness that has progressed rapidly. Checking motor strength on a regular basis to assess muscle weakness and progression of the disease is required. Physical therapy for passive muscle movements to prevent atrophy and contractures is also needed [22]. Monitoring is essential as serious secondary complications can occur within hours. These include respiratory distress requiring intubation, hemodynamic instability, and dysfunction of the autonomic nervous system. 

 IgG infusions and plasma exchange transfusions are the treatments geared toward the immune response of the syndrome. IVIG is easier to give, has fewer side effects, and the outcome is as good as plasmapheresis; therefore, IVIG is the initial treatment of choice. Earlier ambulation and improved score on the Motor Disability Grading Scale (MDGS) was shown with IVIG treatment in the pediatric population. A dose of 1 g/kg daily for 2 days was used [23]. If IVIG therapy fails, however, plasmapheresis is recommended [24]. Studies have shown improvement of patients who receive plasma-exchange transfusions within two weeks of the onset of symptoms. Two and five plasma exchanges are recommended for mild and severe GBS, respectively. In one pediatric trial, no difference in recovery was found when comparing 2 g/kg IVIG given over a 2-day versus a 5-day period. However, the 2 day treatment group had more early relapses [25]. 

 Corticosteroids alone are known to have no benefit in the treatment of GBS. Despite their anti-inflammatory activity and their use in treating other autoimmune diseases, at this time there is no evidence supporting the use of corticosteroids alone or in combination therapy for treating GBS.

### 2.9. Prognosis

 In the pediatric population, one study of 47 patients found that below the age of 9, if the peak of muscle weakness was less than 10 days, there was an increased risk for residual muscle weakness [26]. A pediatric study in Turkey involving 23 patients showed similar overall outcomes at one year when comparing axonal and demyelinating forms of GBS. However, the patients diagnosed with the axonal form of the syndrome have a delayed recovery over the first year when compared to the demyelinating patients [27]. Overall, the pediatric patient with GBS does well and symptoms resolve. 

### 2.10. Summary

 We have presented a case of a 14-month-old female who presented with upper and lower extremity weakness and refusal to walk. She was diagnosed with Guillain-Barre syndrome. Although GBS is rare under two years of age, GBS must be considered with the appropriate presentation. We also have provided a brief review of GBS for the emergency medicine physician. Guillain-Barre syndrome is a neurological medical emergency. Rapid deterioration and weakness leading to respiratory failure and cardiac problems due to autonomic dysfunction require intensive care monitoring. If delayed diagnosis and treatment occur in the emergency department, they can cause more complications and a worse outcome for the patient. Therefore, it is essential for the emergency medicine physician to consider the diagnosis in the pediatric patient presenting with weakness, limping, lower extremity pain, areflexia, or refusal to walk.
